# Ameliorated ConA-Induced Hepatitis in the Absence of PKC-theta

**DOI:** 10.1371/journal.pone.0031174

**Published:** 2012-02-07

**Authors:** Xianfeng Fang, Ruiqing Wang, Jian Ma, Yan Ding, Weirong Shang, Zuoming Sun

**Affiliations:** 1 Division of Immunology, Beckman Research Institute of the City of Hope, Duarte, California, United States of America; 2 Department of Gynecology and Obstetrics, Emory University School of Medicine, Atlanta, Georgia, United States of America; Institute of Hepatology London, United Kingdom

## Abstract

Severe liver injury that occurs when immune cells mistakenly attack an individual's own liver cells leads to autoimmune hepatitis. In mice, acute hepatitis can be induced by concanavalin A (ConA) treatment, which causes rapid activation of CD1d-positive natural killer (NK) T cells. These activated NKT cells produce large amounts of cytokines, which induce strong inflammation that damages liver tissues. Here we show that *PKC-θ^−/−^* mice were resistant to ConA-induced hepatitis due to essential function of PKC-θ in NKT cell development and activation. A dosage of ConA (25 mg/kg) that was lethal to wild-type (WT) mice failed to induce death resulting from liver injury in *PKC-θ^−/−^* mice. Correspondingly, ConA-induced production of cytokines such as IFNγ, IL-6, and TNFα, which mediate the inflammation responsible for liver injury, were significantly lower in *PKC-θ^−/−^* mice. Peripheral NKT cells had developmental defects at early stages in the thymus in *PKC-θ^−/−^* mice, and as a result their frequency and number were greatly reduced. Furthermore, *PKC-θ^−/−^* bone marrow adoptively transferred to WT mice displayed similar defects in NKT cell development, suggesting an intrinsic requirement for PKC-θ in NKT cell development. In addition, upon stimulation with NKT cell-specific lipid ligand, peripheral *PKC-θ^−/−^* NKT cells produced lower levels of inflammatory cytokines than that of WT NKT cells, suggesting that activation of NKT cells also requires PKC-θ. Our results suggest PKC-θ is an essential molecule required for activation of NKT cell to induce hepatitis, and thus, is a potential drug target for prevention of autoimmune hepatitis.

## Introduction

Mistaken attack of healthy liver cells by an individual's own immune system causes severe liver damage, leading to autoimmune hepatitis (AIH) [1]. A widely used murine AIH model is that caused by concanavalin A (ConA) treatment, which rapidly induces severe immune-mediated hepatitis due to activation of a specific population of T cells, natural killer (NK) T cells, that are enriched in liver [2]. Activated NKT cells produce large amounts of inflammatory cytokines such as IFNγ, IL-4, TNFα and MCP1, which in turn recruit innate immune cells such as macrophages to cause inflammatory responses [3], [4]. In addition, activated NKT cells also up-regulate FasL and induce hepatocyte apoptosis through the FasL-Fas pathway. Fas/FasL-mediated apoptosis appears to be an important mechanism for liver damage, as NKT cells from Fas-mutant gld/gld mice fail to induce hepatitis [5], [6]. Although ConA can activate other T cells, NKT cells are required and sufficient for induction of liver damage in this murine AIH model [7]. NKT cells are also thought to be involved in liver injury induced by LPS, α-galactosylceramide (α-GalCer), Salmonella infection, chronic hepatitis C infection and primary biliary cirrhosis [8], [9], [10], [11], [12]. TCR signaling molecules are likely to have an essential role in the activation of NKT cells responsible for hepatitis, as suggested by the prevention of hepatitis by immunosuppressive drugs such as FK506 or cyclosporine, which inhibit conventional T cell receptor (TCR) signals [13]. Thus, critical TCR signaling molecules are potential drug targets for treatment of hepatitis; however, little is known about the signaling molecules required for activation of NKT.

NKT cells develop in the thymus and are positively selected by the MHC-I-like molecule CD1d [14], as indicated by complete absence of NKT cells in CD1d-deficient mice [15]. NKT cell development involves the following sequential stages: stage 0) CD24^hi^; stage 1) CD24^int^CD44^neg^NK1.1^neg^; stage 2) CD44^+^NK1.1^−^ and; stage 3) CD44^+^NK1.1^+^ mature NKT cells [15]. Mature NKT cells express TCRs that consist of an invariant Vα14-Jα18 TCRαβ chain paired with a limited number of TCRβ chains, Vβ8, Vβ7 or Vβ2, which is why they are called invariant NKT (iNKT). TCRs on NKT cells recognize CD1d-presented glycolipids such as α-GalCer, a potent activator of both mouse and human NKT cells [16]. Little is known about the signaling pathways that regulate NKT development; however, the NF-κB pathway is likely important, as a dominant negative IκB transgene can arrest NKT development at the CD44^+^NK1.1^−^ stage [17]. NF-κB is an important downstream signaling molecule of TCR, and therefore is likely that TCR mediates the activation of NF-κB required for NKT development.

PKC-θ mediates the critical TCR signals required for conventional T cell activation [18], [19], [20]. Engagement of TCR induces activation of phospholipase Cγ1 (PLCγ1), which catalyzes the hydrolysis of inositol phospholipids to produce diacylglycerol (DAG) and inositol triphosphate (IP_3_). DAG activates PKCs [21]. Although phorbal esters activate multiple isoforms of PKC, PKC-θ is selectively required for T cell activation *in vivo* [19], [20]. Mature *PKC-θ^−/−^* T cells failed to proliferate and produce interleukin 2 (IL-2) upon TCR stimulation due to defective activation of NF-κB and AP1, and these observations are supported by several *in vitro* studies in Jurkat T cells [22], [23], [24], [25]. Mice deficient in other isoforms of PKC do not display defects similar to those observed in *PKC-θ^−/−^* T cells [26], demonstrating the selective requirement of PKC-θ in T cell activation. Although many T cell-dependent immune disease models have been used to demonstrate PKC-θ regulated T cells function *in vivo* [27], it is unknown how PKC-θ functions in NKT cell-mediated *in vivo* immune responses. In this study, we used ConA-induced hepatitis to define the essential function of PKC-θ in NKT cell-mediated liver injury, strongly suggesting PKC-θ is a potential drug target for the prevention autoimmune hepatitis.

## Materials and Methods

### Mice

All experiments involving mice were approved by the City of Hope Institutional Animal Care and Use Committee. B6-Ly5.2/Cr(CD45.1) mice were purchased from NCI laboratories (Frederick, MD). *PKC-θ^−/−^* mice were generated as previously described [20]. Mice used were in C57BL/6 background and age/sex matched between WT and *PKC-θ^−/−^* mice. All mice were maintained under specific pathogen-free conditions.

### Antibodies and tetramers

Monoclonal antibodies CD3 (clone 145-2c11), NK1.1 (clone PK136), CD44 (clone IM7), CD24 (clone M1/69), CD45.1 (clone A20), CD45.2 (clone 104), IL4 (clone 11B11), INFγ (clone XMG1.2), FasL (clone MFL3) were purchased from eBioscience (San Diego, CA). CD40L(clone MR1), Trail (clone N2B2) and DR5 (clone MD5-1) were purchased from Biolegend (San Diego, CA). The PE-CD1d-PBS157 tetramer was provided by the tetramer core facility at the National Institutes of Health.

### ConA and OCH treatment

ConA (Sigma, St. Louis, MO) was dissolved in pyrogen-free phosphate-buffered saline (PBS), and intravenously injected (25 mg/kg) into wild-type (WT) and *PKC-θ^−/−^* mice via the tail vein. Mouse survival was monitored for 56 h after injection. OCH (provided by the National Institutes of Health), a specific antigen of NKT cells, was injected intravenously (1 µg/mouse) via the tail vein into both WT and *PKC-θ^−/−^* mice. One hour after injection, mice were bled and their sera collected. Mice were then sacrificed for collection of intrahepatic lymphocytes as previously described [28].

### Serum cytokine and ALT/AST assay

Collected sera were 1∶10 diluted with the diluents provided by the mouse inflammation CBA kit (BD Biosciences, San Diego, CA). Inflammatory cytokines (INFγ, TNF, MCP1, IL6) were measured using the mouse inflammation CBA kit. Mouse IL-4 was assayed with the flex set of cytokine assay beads (BD Biosciences). Mouse serum ALT and AST were assayed with the enzymatic assay kit (Bioo Scientific, Austin, TX). Mouse serum Osteopontin (OPN) were assayed with Osteipontin mouse ELISA Kit (Abcam, Cambridge, MA).

### Surface and intracellular staining

Cells were incubated (30 min, 4°C, in the dark) with antibody cocktail in staining buffer (2% FBS plus 0.1% sodium azide). Cell samples were then washed and examined by BD FACSCanto II (BD Biosciences). For intracellular staining, subsequent to surface staining, cells were fixed with BD Cyto fix/perm buffer for 15 min followed by two washes with Cyto perm/wash buffer. Cells were then incubated (20 min, 4°C, in the dark) with IL-4/INFγ cocktail in Cyto/perm buffer. After two washes, cells were examined by FACSCanto II. The FACS data were analyzed with Flowjo 7.4.6 (Tree Star).

### Generation of bone marrow chimeric mice

Bone marrow (BM) transfer was performed as described [29]. Briefly, WT and *PKC-θ^−/−^* mice received whole body γ-irradiation (two doses of 550 Rad given 3 h apart) with a cesium source (Gammacell 40), and the bone marrow recipient mice were reconstituted 6 h later with one intravenous injection of 5×10^6^ bone marrow cells from various adult donors. After 10 weeks of reconstitution, mice thymus NKT cells were analyzed.

### FACS sorting of NKT cells and *in vitro* stimulation

Pooled splenocytes and intrahepatic lymphocytes from batches of two WT and two *PKC-θ^−/−^* mice were stained with anti-CD3 antibody and CD1d-PBS157 tetramer as above, and the NKT cells (CD3^low^CD1d-PBS157 tetramer^+^ subset) sorted by BD FACSAria III (BD Biosciences). The purified NKT cells were cultured in 96 well plates (2.5×10^5^ cells/well) with 200 µl/well T cell culture medium (RPMI 1640 supplemented with 10% fetal calf serum, 5×10^−5^ M 2-mercaptoethanol, 2 mM L-glutamine, 1 mM sodium pyruvate, 0.1 mM non-essential amino acids, 100 U/ml penicillin and 100 µg/ml streptomycin) with or without 100 ng/ml OCH as previously described [30]. After overnight stimulation, cytokine levels in supernatants were assayed with BD Flex set, and cells were harvested for IL4 and IFNγ intracellular staining as above.

### Statistical analysis

Prism software (Graphpad) was used for all statistical analyses. Unpaired students t tests were used to compare experimental groups. A P value of less than 0.05 was considered statistically significant.

## Results

### *PKC-θ^−/−^* mice are resistant to ConA-induced hepatitis

**Figure 1:**
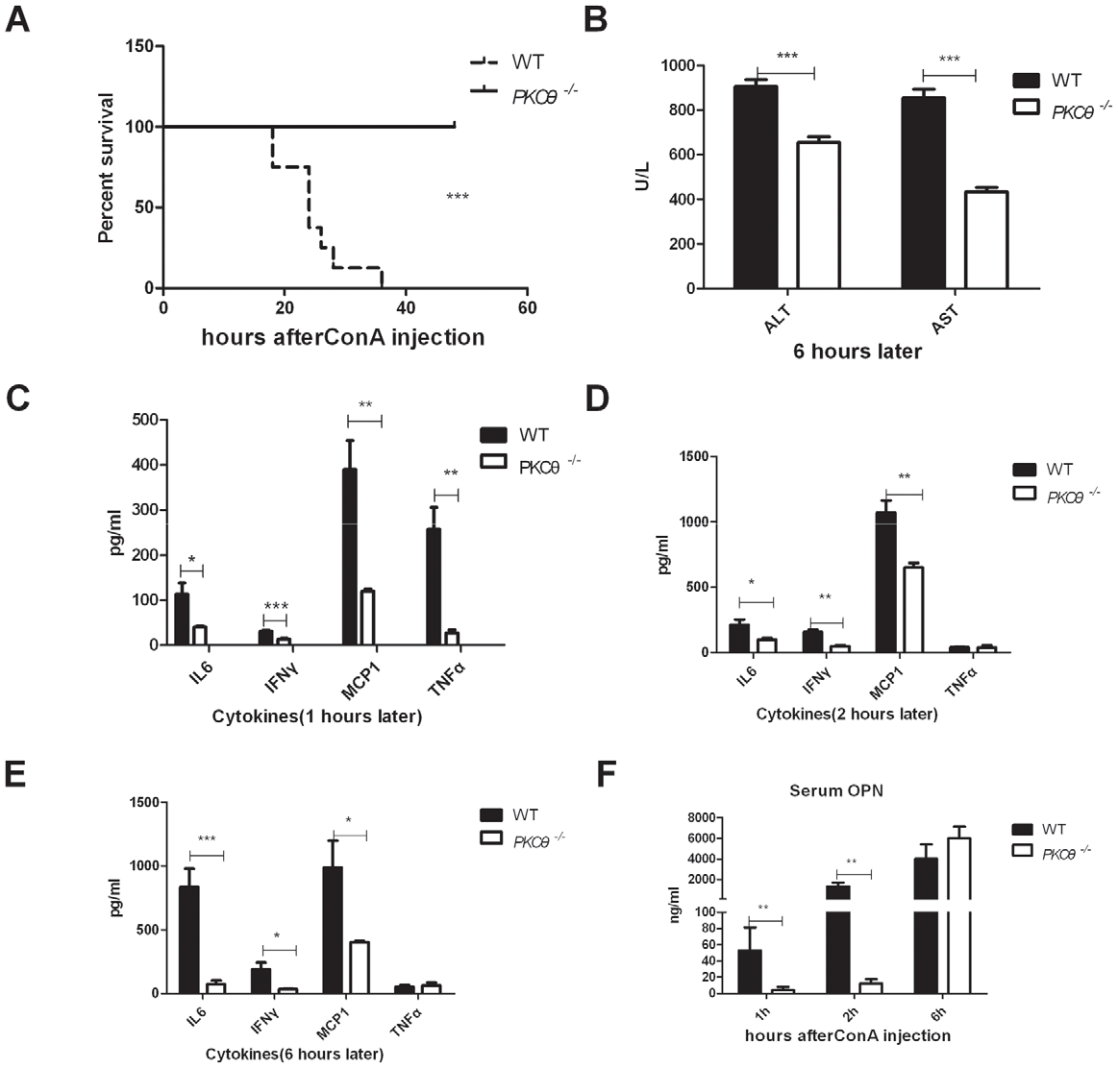
*PKC-θ^−/−^* mice are resistant to ConA-induced hepatitis. *PKC-θ^−/−^* mice and age and sex matched WT mice were challenged with a lethal dosage of ConA (25 mg/kg). Survival was monitored every 3 h. N = 7, from 2 independent experiments. A) Survival curve of mice challenged with ConA. B) Serum ALT and AST levels 6 h after ConA challenge. C) Levels of the indicated cytokines in serum 1 h after ConA challenge. D) Levels of the indicated cytokines in serum 2 h after ConA challenge. E) Levels of the indicated cytokines in serum 6 h after ConA challenge. F) Levels of serum OPN different times after ConA challenge. (*, P<0.05; **, P<0.01; ***, P<0.001).

To determine the function of PKC-θ in liver injury, we used an acute hepatitis murine model that depends on ConA-mediated activation of NKT cells [31]. WT and PKC-θ^−/−^ mice, age and sex matched, were treated with 25 mg/kg ConA, and their survival rate was determined. Consistent with previous results [9], this dosage of ConA was lethal for WT mice (Fig. 1A), whereas all *PKC-θ^−/−^* mice survived. Because damaged liver releases aspartate transaminase (AST) and alanine transaminase (ALT), we measured levels of both enzymes in serum of ConA-treated mice to assess liver damage (Fig. 1B). Prior to ConA treatment, AST and ALT levels were both very low (less than 50 U/L), and there were no obvious differences between WT and *PKC-θ^−/−^* mice (data not shown). After ConA treatment, although elevated, AST and ALT levels of PKC-θ^−/−^ mice were significantly lower than those of WT mice, suggesting there was less liver damage in the absence of PKC-θ. Because ConA treatment stimulates the production of inflammatory cytokines that are critical mediators for liver injury, we also measured serum cytokines after ConA treatment. Levels of the inflammatory cytokines IL-6, IFNγ and monocyte chemotactic protein-1 (MCP1) were significantly lower in *PKC-θ^−/−^* than WT mice at 1 h (Fig. 1C), 2 h (Fig. 1D) and 6 h (Fig. 1E) after ConA treatment. TNFα peak was detected at 1 h after stimulation in WT mice, which is consistent with published results [32]. However, there was no such peak of TNFα detected in *PKC-θ^−/−^* mice. We also checked the serum levels of OPN, which has important role in the hepatic inflammation and toxicity [33], after challenged with ConA at different time point (Fig. 1F). *PKC-θ^−/−^* mice had lower levels of OPN in the early stage after ConA challenge (1 h and 2 h). However, there was no obvious difference in OPN levels between WT and *PKC-θ^−/−^* mice 6 h after ConA treatment, which is similar to TNFα. These results suggest that PKC-θ is required for ConA-induced inflammation that is responsible for the liver injury.

### *PKC-θ^−/−^* mice have significantly fewer NKT cells

**Figure 2:**
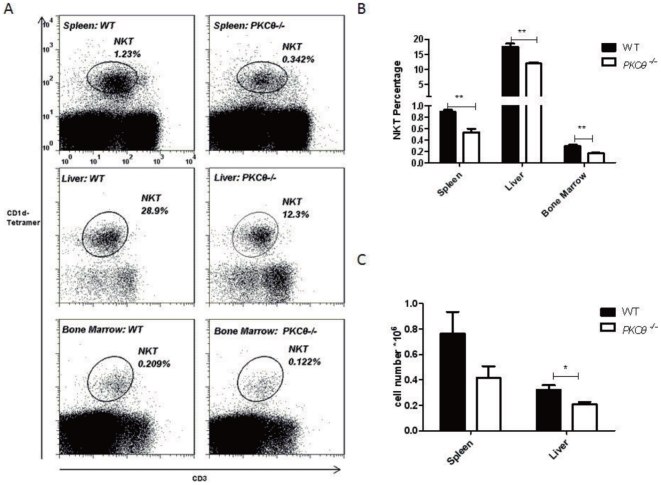
Reduced numbers of NKT cells in peripheral lymphoid tissues of *PKC-θ^−/−^* mice. A) Flow cytometric analysis of CD1d-Tetramer-positive and CD3-positive NKT cells mice in spleen (top panels), liver (middle panels) and bone marrow (bottom panels) of naïve WT (left panels) and *PKC-θ^−/−^* (right panels) mice. B) Frequency of NKT cells in organs described in A, as averaged from five mice of each genotype. C) Total NKT cell number in spleen and liver as averaged from five mice of each genotype. (*, P<0.05; **, P<0.01).

Since liver NKT cells are essential for ConA-induced hepatitis, we examined NKT cell distributions in naïve WT and *PKC-θ^−/−^* mice. Consistent with their role in liver injury [15], NKT cells were enriched in liver tissues (28.9%) as compared to spleen (1.23%) or bone marrow (0.21%) (Fig. 2A, left panel). The percentage of NKT cells in liver (12.3%), spleen (0.342%) and bone marrow (0.122%) of *PKC-θ^−/−^* mice was always lower than in WT mice (Fig. 2A, B). Furthermore, the absolute number of NKT cells in spleen and liver were also significantly less in PKC-θ^−/−^ mice (Fig. 2C), indicating that in the absence of PKC-θ there is a reduction in peripheral NKT cells, which are required for ConA-induced hepatitis.

### NKT cell development is defective in *PKC-θ^−/−^* mice

**Figure 3:**
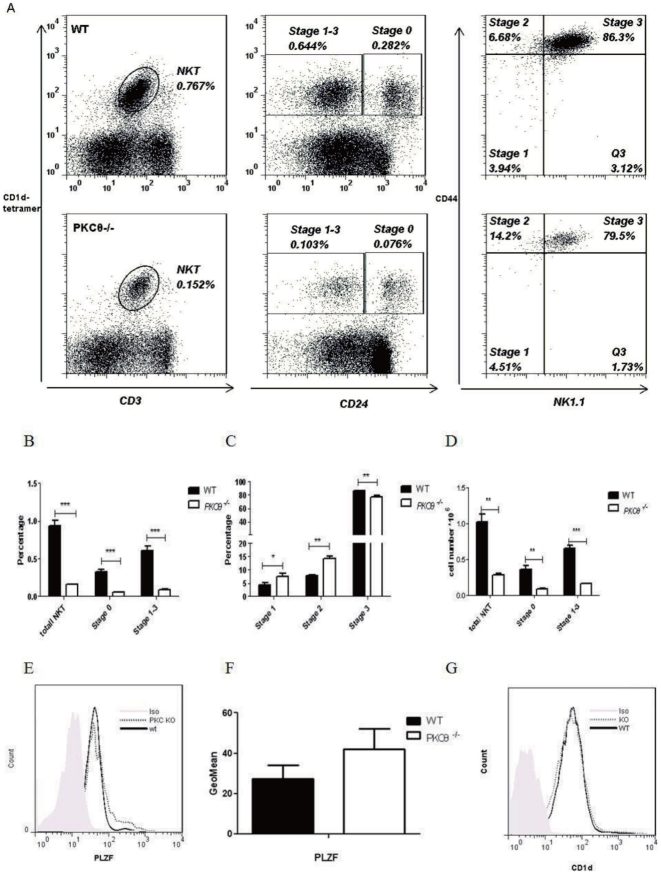
NKT cell development is defective in *PKC-θ^−/−^* mice. A) Flow cytometric analysis of thymic NKT cells in WT (top panels) and *PKC-θ^−/−^* (bottom panels) mice. Overall CD1d- and CD3-positive NKT cells in thymus were first analyzed (left panels), and the NKT cells were then divided into stage 0 and stage 1–3 based on expression of CD24 (middle panels). Gated Stage 1–3 cells were further analyzed based on CD44 and NK1.1 expression to indicate each of the three stages (right panels). B) Frequency of NKT cells in thymus, stage 0 and stage 1–3 described in A, as averaged from three mice of each genotype. C) Frequency of NKT cells at stages 1, 2 and 3 described in A, as averaged from three mice of each genotype. D) Total NKT cell number in thymus as well as stage 0 and stage 1–3, as averaged from three mice (*, P<0.05; **, P<0.01; ***, P<0.001). E) FACS analysis of PLZF expression in thymic NKT cells. PLZF expression in WT and *PKC-θ^−/−^* NKT cells was analyzed using flow cytometry. F) GeoMean of PLZF expression averaged from 3 independent experiments described in E. G) Lack of PKC-θ does not affect surface CD1d levels on thymocytes. Histogram of CD1d levels of WT (black line) and *PKC-θ^−/−^* (dotted line) thymocytes.

That the number of peripheral NKT cells was reduced in all *PKC-θ^−/−^* tissues examined raised the possibility that NKT cell development was inadequate. Since NKT cells develop in the thymus, we assessed the thymic NKT cells. Consistent with results for the periphery, significantly fewer NKT cells (percentage and absolute number) were present in the thymi of *PKC-θ^−/−^* mice (Fig. 3A–D), suggesting abnormal NKT cell development. NKT cell development can be divided into two major stages, stage 0 and stage 1–3, based on expression of the surface marker CD24 [34]. The percentages of NKT cells in both stages were much lower in *PKC-θ^−/−^* mice (Fig. 3B), suggesting defective NKT development at early stages. We further analyzed stage 1–3 cells with the surface markers CD44 and NK1.1 (Fig. 3A, C). *PKC-θ^−/−^* mice accumulated significantly more cells at stages 1 and 2 (4.51% and 14.2%, respectively) than did WT (3.94% and 6.68%), suggesting a blockade at these two stages.

Since PLZF (promyelocytic leukemia zinc finger, Zbtb16) is critical for NKT cell development and function [34], its expression was thus detected by intracellular staining (Fig. 3E–F). PLZF levels in *PKC-θ^−/−^* NKT cells were not lower, actually were slightly higher than WT NKT cells if there was any change, suggesting that PLZF cannot explain the observed defective NKT development in *PKC-θ^−/−^* mice. In addition we examined levels of CD1d which is required to present antigens for positive selection of NKT cells in thymus [14]. No obvious differences in CD1d expression were observed between WT and *PKC-θ^−/−^* thymocytes (Fig. 3G), suggesting that defective NKT cell development in *PKC-θ^−/−^* mice is likely not due to abnormal expression of CD1d. Taken together, our results strongly suggest that PKC-*θ* is required for NKT cell development in thymus, which likely explains the reduction in peripheral NKT cells in *PKC-θ^−/−^* mice.

### PKC-θ is intrinsically required for NKT cell development

**Figure 4:**
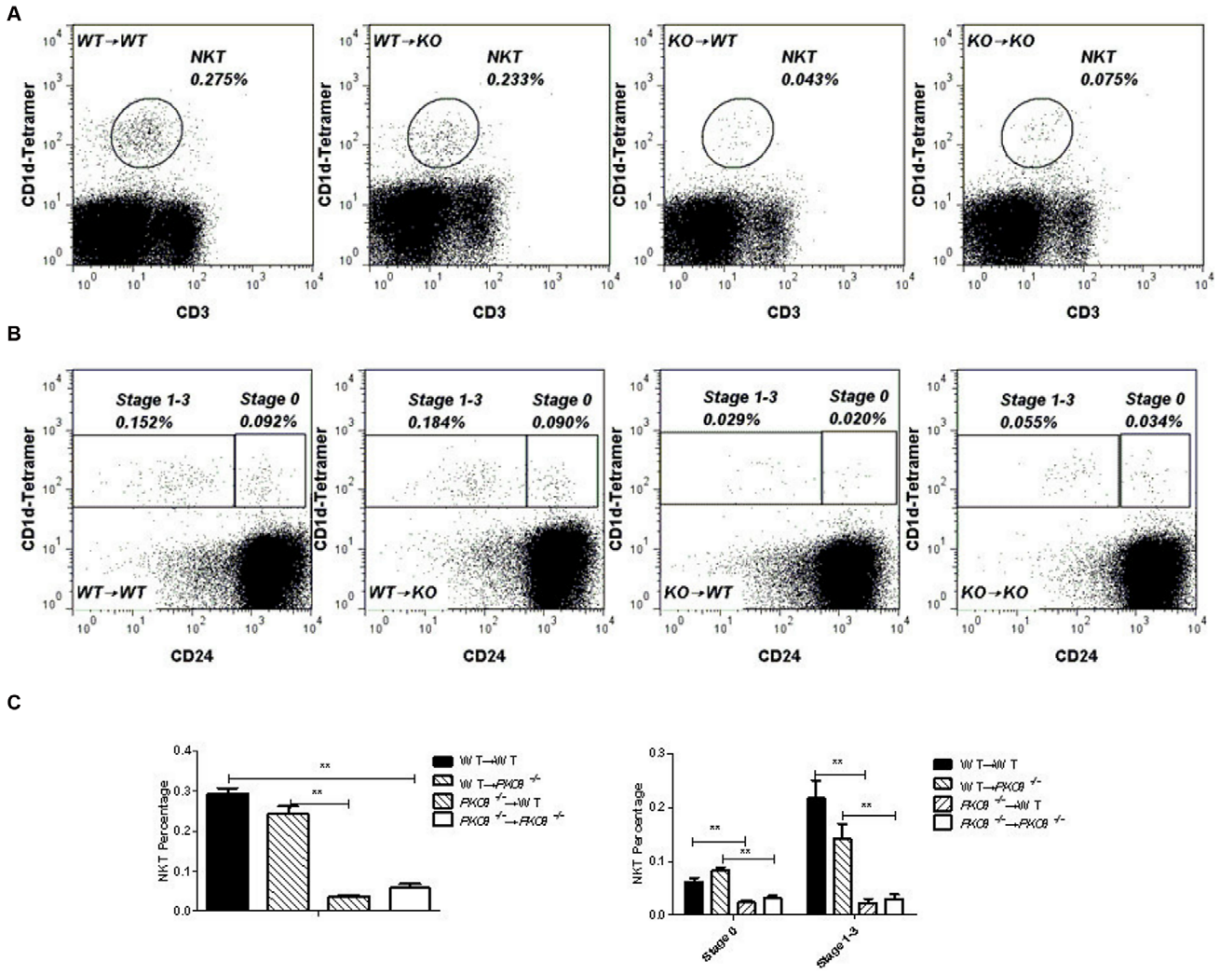
Intrinsic requirement of PKC-θ for NKT cell development. Bone marrow from WT and *PKC-θ^−/−^* donors was adoptively transferred to irradiated WT or *PKC-θ^−/−^* recipients (6 mice per group). Ten weeks after adoptive transfer, NKT cells in thymi were analyzed by flow cytometry. A) CD1d and CD3 NKT cells in thymi of adoptively transferred mice. B) Stage 0 and stage 1–3 NKT cells in thymi of adoptively transferred mice. C) Frequencies of total NKT (left panel), and stage 0 and stage 1–3 NKT cells (right panel) as averaged from 6 recipients of each type of adoptive transfer. (**, P<0.01).

Intact *PKC-θ^−/−^* mice lack PKC-θ in all tissues. Therefore, to determine whether defective NKT cell development is due to lack of PKC-θ in hematopoietic cells or the surrounding tissues, we performed adoptive transfer to examine NKT cell development arising from *PKC-θ^−/−^* bone marrow in a WT environment. We used an congenic marker, CD45.1 (donors) and CD45.2 (WT recipients), to differentiate between donor and recipient cells, and used four different adoptive transfer scenarios: WT (CD45.1) bone marrow to WT (CD45.2) recipients (WT→WT), *PKC-θ^−/−^* bone marrow to WT (CD45.1) recipients (KO→WT), WT (CD45.1) bone marrow to *PKC-θ^−/−^* recipients (WT→KO), and *PKC-θ^−/−^* bone marrow to *PKC-θ^−/−^* recipients (KO→KO). Ten weeks after bone marrow transfer, we examined thymic NKT cells (Fig. 4A and left panel of C) and NKT cells at stages 0 and 1–3 (Fig. 4B and right panel of C). Similar numbers of NKT cells developed from WT bone marrow in WT or *PKC-θ^−/−^* recipients, suggesting that NKT cells can develop normally in a *PKC-θ^−/−^* environment. In contrast, *PKC-θ^−/−^* bone marrow developed much fewer NKT cells in either WT or *PKC-θ^−/−^* recipients, demonstrating that *PKC-θ^−/−^* bone marrow failed to fully reconstitute the NKT cell compartment, even in the WT environment (Fig. 4A, B). Thus, these data show that PKC-θ is intrinsically required for thymic NKT cell development.

### *PKC-θ^−/−^* NKT cells are defective in production of inflammatory cytokines

**Figure 5:**
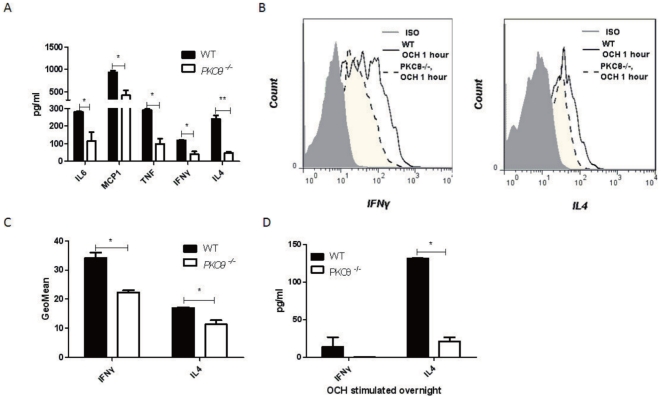
NKT cells require PKC-θ in order to produce cytokines. A) WT (black bars) and *PKC-θ^−/−^* (open bars) mice were challenged with OCH (1 µg) for 1 h, indicated cytokine levels in serum were detected (*, P<0.05; **, P<0.01). B. Mice were challenged with OCH (1 µg) for 1 h, IFNγ(left panel) and IL-4 (left panel) in WT (solid line) and *PKC-θ^−/−^* (dotted line) NKT cells were detected by intracellular staining of intrahepatic lymphocytes. Shaded area is isotype (ISO) antibody control. C) Intracelluar IFNγ and IL-4 levels in NKT cells described in B are indicated by GeoMean obtained from at least three independent experiments. D) Sorted NKT cells (CD3^Low^CD1d-PBS57^+^) were stimulated with 100 ng/ml OCH *in vitro* for overnight, and IFNγ and IL-4 levels in supernatant were measured (*, P<0.05; **, P<0.01).

In response to activation, NKT cells produce cytokines that mediate inflammatory responses to cause liver injury. We have shown that *PKC-θ^−/−^* mice produced lower levels of inflammatory cytokines in response to ConA treatment (Fig. 1). However, ConA can stimulate conventional T cells in addition to NKT cells. Therefore, we examined cytokine production in response to stimulation with OCH, a glycolipid ligand that binds to CD1d and is specific for activation of NKT cells [15]. At 1 h post OCH treatment, IL-6, MCP1, TNFα, INFγ and IL-4 levels in serum were all consistently much lower in *PKC-θ^−/−^* mice (Fig. 5A). To specifically measure cytokines produced by NKT cells, we performed intracellular staining of IFNγ and IL-4 in liver NKT cells 1 h after OCH treatment (Fig. 4B–C). Intrahepatic lymphocytes were collected based on the method previously described [28]. Indeed, *PKC-θ^−/−^* NKT cells had reproducible reduced levels of both IFNγ (Fig. 5B left panel and Fig. 5C) and IL-4 (Fig. 5B right panel and Fig. 5C). Consistently, IFNγ and IL-4 production was also lower in OCH-stimulated purified *PKC-θ^−/−^* NKT cells compared to the WT cells (Fig. 5D). These results thus suggest that PKC-θ is required to activate NKT cells to produce inflammatory cytokines.

### *PKC-θ^−/−^* NKT up-regulate FasL, TRAIL and CD40L normally

**Figure 6:**
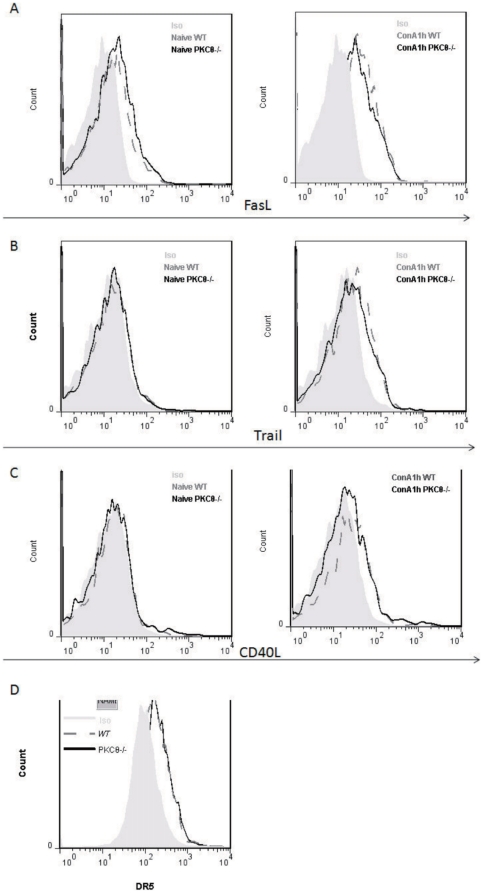
Normal up-regulation of FasL, CD40L and Trail in *PKC-θ^−/−^* mice. Flow cytometric analysis of surface FasL levels (A), CD40L levels (B) and Trail levels (C) of WT (dark grey dash line) and *PKC-θ^−/−^* (black line) NKT cells that were naive (left panel) or stimulated with ConA (15 mg/kg) for 1 h (right panel). D) DR5 expression on WT (dark grey dash line) and *PKC-θ^−/−^* (black line) hepatocytes, Shaded area is isotype (ISO) antibody control.

Upon activation, NKT cells up-regulate FasL, TRAIL and CD40L, which contributes to ConA-induced hepatitis by induction apoptosis [5], [6], [35], [36]. Therefore, we examined their expression after ConA stimulation (Fig. 6). There were no obvious difference in FasL (Fig. 6A), Trail (Fig. 6B) and CD40L (Fig. 6C) levels on NKT cells between WT and *PKC-θ^−/−^* mice before (left panels) or after (right panels) ConA challenge, although stimulation up-regulated their levels as expected. In addition, examination of expression of DR5 (Fig. 6D), the ligand for Trail, on hepatocytes were found no difference between WT and *PKC-θ^−/−^* mice. These results suggest that it is unlikely that FasL, Trail and CD40L contribute to the hepatitis-resistance exhibited by *PKC-θ^−/−^* mice.

## Discussion

Liver diseases, including acute liver failure, viral hepatitis, alcoholic liver disease, biliary cirrhosis and AIH, are serious threats to public health. Although NKT cells represent only about 0.5% of total thymocytes and peripheral T cells, they comprise up to 30% of the T cells in liver and play a critical role in liver diseases [37]. Overwhelming activation of NKT cells, such as that induced by ConA, α-GalCer, LPS and salmonella infection, causes much damage, including severe injury of the liver [4], [9], [15]. ConA-induced hepatitis has been used as a model for NKT-mediated liver injury, which is closely resembles the pathology of human AIH [4]. Inhibition of NKT cell activation is thus beneficial under such circumstances. We found that PKC-θ is a critical molecule required for NKT cell activation by ConA and its lipid ligand, and that deletion of PKC-θ likely impairs liver injury in AIH. Many highly specific PKC-θ inhibitors have been developed by pharmaceutical companies [38], [39], and these inhibitors likely have therapeutic value in the treatment of AIH.

Lack of PKC-θ interferes with multiple NKT cell functions that contribute to the ameliorated ConA-induced hepatitis observed in *PKC-θ^−/−^* mice. First, both the percentage of NKT cells and their absolute number were reduced in liver. This reduction in NKT cells is not restricted to liver, as reduced numbers of NKT cells were also observed in the spleen, bone marrow and thymus. That numbers of thymic NKT cells were significantly reduced suggests that development of NKT cells requires PKC-θ, which is also confirmed by other observations [40], [41]. However, it was not clear at which stages NKT cell development was blocked. Our results demonstrated a significant reduction in NKT cells starting from the earliest developmental stages (stage 0), suggesting PKC-θ has a critical function during early NKT development. Further analysis of stages 1–3 showed that lack of PKC-θ lead to block NKT cell development at stages 1 and 2 but not so much at stage 3, suggesting that PKC-θ-mediated function is more important for stage 1 and 2 NKT cell development than that of stage 3. We have shown previously that PKC-θ regulates NF-κB activation [20], and NF-κB is apparently required for NKT development [17]. However, deletion of NF-κBp50 arrested NKT cell development at stage 2, which is a later stage than what we observed in *PKC-θ^−/−^* mice. In addition to the NF-κB pathway, we and others have shown that PKC-θ also regulates signaling pathways such as AP1 and NFAT [42]. It is likely that PKC-θ-regulated AP1 or NFAT or both may play a role in early stages of NKT cell development. The critical role of PKC-θ in NKT development is in contrast to conventional T cells, in which development is largely independent of PKC-θ [20], and it remains to be determined why PKC-θ is specifically required for the development of NKT cells.

In addition to reduced NKT cell number, defective NKT cell activation also contributes to ameliorated hepatitis in *PKC-θ^−/−^* mice. Inflammatory cytokines such as IFNγ, IL-4 and TNFα that are produced by activated NKT cells are essential mediators for induction of hepatitis [4], [17], [31]. We found that NKT cells activated by OCH in the absence of PKC-θ produced much less IFNγ, IL-4 and TNFα. Consistent with this, fewer NKT cells from *PKC-θ^−/−^* mice produced IFNγ and IL-4 *in vitro* and *in vivo*, suggesting that reduced total number of NKT cells likely contributes to the reduced serum TNFα. Interestingly, upon ConA treatment, TNFα, which was lower in *PKC-θ^−/−^* mice 1 hr after stimulation, but has no obvious difference 2 hr and 6 hr after stimulation. It is important to emphasize here that other cells such as macrophages also produce TNFα upon activation. Therefore, it is possible that TNFα produced by other cells may contribute to the increased TNFα levels in serum. It appears that IL-4 can regulate TNFα production in ConA-induced hepatitis, as exogenous IL-4 can boost TNFα levels [43]. Therefore, the reduced IL-4 production by *PKC-θ^−/−^* NKT cells may also be responsible for lower levels of TNFα in the serum. Here we have demonstrated that PKC-θ mediates signals required for IFNγ and IL-4 production by NKT cells, which complements our previous results showing that PKC-γ mediates critical TCR signals required for IL-2 production in T cells [20]. In addition to IFNγ and IL-4, osteopontin produced by activated NKT also contributes to ConA-induced hepatitis, indicated by the resistance to ConA-induced hepatitis by osteopontin-deficient mice [44]. Since *PKC-θ^−/−^* mice have reduced NKT cell number and impaired NKT cell activation, it is likely that osteopontin produced by activated NKT cells is also correspondingly reduced in the absence of PKC-θ, which contributes to the observed impaired hepatitis in *PKC-θ^−/−^* mice.

Another potential mechanism responsible for NKT cell-induced liver injury is FasL-induced hepatocyte apoptosis [5], [6], [35], [36]. Similar to conventional T cells, activation of NKT cells leads to up-regulation of FasL, which interacts with Fas on surface of hepatocytes and induces apoptosis. The Fas-FasL-mediated apoptosis is essential for ConA-induced hepatitis, as mutations in this apoptotic pathway prevent hepatitis [5], [6]. We have previously shown that PKC-θ is required for up-regulation of FasL in conventional T cells and Fas/FasL-mediated activation-induced cell death [45]. To our surprise, FasL was normally up-regulated in NKT cells in the absence of PKC-θ. Similarly, we also did not find obvious difference in TRAIL, CD40, expression between WT and *PKC-θ^−/−^* NKT cells. CD40 expression on hepatocytes is also not affected by deletion of PKC-θ (data not shown). Therefore, in contrast to other functions such as activation and cytokine production, PKC-θ plays a different function in conventional T than in NKT cells during FasL and likely TRAIL and CD40 up-regulation.

In summary, *PKC-θ^−/−^* mice are resistant to ConA-induced hepatitis, and this resistance is due to at least following mechanisms: 1) reduced NKT cell number due to an intrinsic requirement of PKC-θ for NKT cell development, and 2) reduced levels of inflammatory cytokines such as IFNγ and IL-4, because PKC-θ mediates the critical signals required for NKT activation. AIH in humans is generally thought to be an immune disease. Similar to many other autoimmunity, women are affected more than men (gender ratio, 3.6∶1) [46]. Immunosuppressive corticosteroid treatment of AIH is effective [47]; however, corticosteroids have broad effects on many tissues and have potential serious side effects such as bone and skin problems as well as high blood pressure. Highly specific drugs that target T cells, including NKT cells, are likely better candidates for preventing liver injury induced by AIH. PKC-θ is specifically expressed in hematopoietic cells, particularly in T cells, and deletion of PKC-θ specifically affects T cell function [20]. Therefore, many pharmaceutical companies have developed PKC-θ inhibitors to treat T cell-mediated autoimmunity. Consistent with this, our study strongly suggests that PKC-θ is a valuable drug target for treatment of AIH.

## References

[1] McFarlane IG (1999). Pathogenesis of autoimmune hepatitis.. Biomed Pharmacother.

[2] Emoto M, Kaufmann SH (2003). Liver NKT cells: an account of heterogeneity.. Trends Immunol.

[3] Faggioni R, Jones-Carson J, Reed DA, Dinarello CA, Feingold KR (2000). Leptin-deficient (ob/ob) mice are protected from T cell-mediated hepatotoxicity: role of tumor necrosis factor alpha and IL-18.. Proc Natl Acad Sci U S A.

[4] Gantner F, Leist M, Lohse AW, Germann PG, Tiegs G (1995). Concanavalin A-induced T-cell-mediated hepatic injury in mice: the role of tumor necrosis factor.. Hepatology.

[5] Leist M, Gantner F, Bohlinger I, Tiegs G, Germann PG (1995). Tumor necrosis factor-induced hepatocyte apoptosis precedes liver failure in experimental murine shock models.. Am J Pathol.

[6] Gumperz JE, Brenner MB (2001). CD1-specific T cells in microbial immunity.. Curr Opin Immunol.

[7] Godfrey DI, Hammond KJ, Poulton LD, Smyth MJ, Baxter AG (2000). NKT cells: facts, functions and fallacies.. Immunol Today.

[8] Ishigami M, Nishimura H, Naiki Y, Yoshioka K, Kawano T (1999). The roles of intrahepatic Valpha14(+) NK1.1(+) T cells for liver injury induced by Salmonella infection in mice.. Hepatology.

[9] Kaneko Y, Harada M, Kawano T, Yamashita M, Shibata Y (2000). Augmentation of Valpha14 NKT cell-mediated cytotoxicity by interleukin 4 in an autocrine mechanism resulting in the development of concanavalin A-induced hepatitis.. J Exp Med.

[10] Kato A, Yoshidome H, Edwards MJ, Lentsch AB (2000). Regulation of liver inflammatory injury by signal transducer and activator of transcription-6.. Am J Pathol.

[11] Kawano T, Nakayama T, Kamada N, Kaneko Y, Harada M (1999). Antitumor cytotoxicity mediated by ligand-activated human V alpha24 NKT cells.. Cancer Res.

[12] Kim CH, Butcher EC, Johnston B (2002). Distinct subsets of human Valpha24-invariant NKT cells: cytokine responses and chemokine receptor expression.. Trends Immunol.

[13] Tiegs G, Hentschel J, Wendel A (1992). A T cell-dependent experimental liver injury in mice inducible by concanavalin A.. J Clin Invest.

[14] Bendelac A (1995). Positive selection of mouse NK1+ T cells by CD1-expressing cortical thymocytes.. J Exp Med.

[15] Bendelac A, Savage PB, Teyton L (2007). The biology of NKT cells.. Annu Rev Immunol.

[16] Crowe NY, Uldrich AP, Kyparissoudis K, Hammond KJ, Hayakawa Y (2003). Glycolipid antigen drives rapid expansion and sustained cytokine production by NK T cells.. J Immunol.

[17] Sivakumar V, Hammond KJ, Howells N, Pfeffer K, Weih F (2003). Differential requirement for Rel/nuclear factor kappa B family members in natural killer T cell development.. J Exp Med.

[18] Altman A, Isakov N, Baier G (2000). Protein kinase Ctheta: a new essential superstar on the T-cell stage.. Immunol Today.

[19] Pfeifhofer C, Kofler K, Gruber T, Tabrizi NG, Lutz C (2003). Protein kinase C theta affects Ca2+ mobilization and NFAT cell activation in primary mouse T cells.. J Exp Med.

[20] Sun Z, Arendt CW, Ellmeier W, Schaeffer EM, Sunshine MJ (2000). PKC-theta is required for TCR-induced NF-kappaB activation in mature but not immature T lymphocytes.. Nature.

[21] Weiss A, Littman DR (1994). Signal transduction by lymphocyte antigen receptors.. Cell.

[22] Baier-Bitterlich G, Uberall F, Bauer B, Fresser F, Wachter H (1996). Protein kinase C-theta isoenzyme selective stimulation of the transcription factor complex AP-1 in T lymphocytes.. Mol Cell Biol.

[23] Coudronniere N, Villalba M, Englund N, Altman A (2000). NF-kappa B activation induced by T cell receptor/CD28 costimulation is mediated by protein kinase C-theta.. Proc Natl Acad Sci U S A.

[24] Lin X, O'Mahony A, Mu Y, Geleziunas R, Greene WC (2000). Protein kinase C-theta participates in NF-kappaB activation induced by CD3–CD28 costimulation through selective activation of IkappaB kinase beta.. Mol Cell Biol.

[25] Li Y, Hu J, Vita R, Sun B, Tabata H (2004). SPAK kinase is a substrate and target of PKCtheta in T-cell receptor-induced AP-1 activation pathway.. EMBO J.

[26] Baier G (2003). The PKC gene module: molecular biosystematics to resolve its T cell functions.. Immunol Rev.

[27] Kwon MJ, Wang R, Ma J, Sun Z (2010). PKC-theta is a drug target for prevention of T cell-mediated autoimmunity and allograft rejection.. Endocr Metab Immune Disord Drug Targets.

[28] Fang X, Du P, Liu Y, Tang J (2010). Efficient Isolation of Mouse Liver NKT Cells by Perfusion.. PLoS ONE.

[29] Spangrude GJ (2008). Assessment of lymphocyte development in radiation bone marrow chimeras.. Curr Protoc Immunol Chapter.

[30] Watarai H, Nakagawa R, Omori-Miyake M, Dashtsoodol N, Taniguchi M (2008). Methods for detection, isolation and culture of mouse and human invariant NKT cells.. Nat Protocols.

[31] Nicoletti F, Zaccone P, Xiang M, Magro G, Di Mauro M (2000). Essential pathogenetic role for interferon (IFN-)gamma in concanavalin A-induced T cell-dependent hepatitis: exacerbation by exogenous IFN-gamma and prevention by IFN-gamma receptor-immunoglobulin fusion protein.. Cytokine.

[32] van Deuren M (1994). Kinetics of tumour necrosis factor-alpha, soluble tumour necrosis factor receptors, interleukin 1-beta and its receptor antagonist during serious infections.. Eur J Clin Microbiol Infect Dis.

[33] Ramaiah SK, Rittling S (2008). Pathophysiological role of osteopontin in hepatic inflammation, toxicity, and cancer.. Toxicol Sci.

[34] Savage AK, Constantinides MG, Han J, Picard D, Martin E (2008). The transcription factor PLZF directs the effector program of the NKT cell lineage.. Immunity.

[35] Higuchi H, Grambihler A, Canbay A, Bronk SF, Gores GJ (2004). Bile acids up-regulate death receptor 5/TRAIL-receptor 2 expression via a c-Jun N-terminal kinase-dependent pathway involving Sp1.. J Biol Chem.

[36] Malhi H, Barreyro FJ, Isomoto H, Bronk SF, Gores GJ (2007). Free fatty acids sensitise hepatocytes to TRAIL mediated cytotoxicity.. Gut.

[37] Exley MA, Koziel MJ (2004). To be or not to be NKT: natural killer T cells in the liver.. Hepatology.

[38] Mosyak L, Xu Z, Joseph-McCarthy D, Brooijmans N, Somers W (2007). Structure-based optimization of PKCtheta inhibitors.. Biochem Soc Trans.

[39] Cywin CL, Dahmann G, Prokopowicz AS, Young ER, Magolda RL (2007). Discovery of potent and selective PKC-theta inhibitors.. Bioorg Med Chem Lett.

[40] Stanic AK, Bezbradica JS, Park JJ, Van Kaer L, Boothby MR (2004). Cutting edge: the ontogeny and function of Va14Ja18 natural T lymphocytes require signal processing by protein kinase C theta and NF-kappa B.. J Immunol.

[41] Schmidt-Supprian M, Tian J, Grant EP, Pasparakis M, Maehr R (2004). Differential dependence of CD4+CD25+ regulatory and natural killer-like T cells on signals leading to NF-kappaB activation.. Proc Natl Acad Sci U S A.

[42] Manicassamy S, Gupta S, Sun Z (2006). Selective function of PKC-theta in T cells.. Cell Mol Immunol.

[43] Toyabe S, Seki S, Iiai T, Takeda K, Shirai K (1997). Requirement of IL-4 and liver NK1+ T cells for concanavalin A-induced hepatic injury in mice.. J Immunol.

[44] Diao H, Kon S, Iwabuchi K, Kimura C, Morimoto J (2004). Osteopontin as a mediator of NKT cell function in T cell-mediated liver diseases.. Immunity.

[45] Manicassamy S, Sun Z (2007). The critical role of protein kinase C-theta in Fas/Fas ligand-mediated apoptosis.. J Immunol.

[46] Czaja AJ, dos Santos RM, Porto A, Santrach PJ, Moore SB (1998). Immune phenotype of chronic liver disease.. Dig Dis Sci.

[47] Czaja AJ, Freese DK (2002). Diagnosis and treatment of autoimmune hepatitis.. Hepatology.

